# Human Intestinal Organoids and Microphysiological Systems for Modeling Radiotoxicity and Assessing Radioprotective Agents

**DOI:** 10.3390/cancers15245859

**Published:** 2023-12-15

**Authors:** Eloïse Bouges, Charlotte Segers, Natalie Leys, Sarah Lebeer, Jianbo Zhang, Felice Mastroleo

**Affiliations:** 1 RadioPharma Research, Nuclear Medical Applications, Belgian Nuclear Research Centre (SCK CEN), Boeretang 200, 2400 Mol, Belgium; eloise.bouges@sckcen.be (E.B.); charlotte.segers@sckcen.be (C.S.); natalie.leys@sckcen.be (N.L.); 2 Department of Bioscience Engineering, University of Antwerp, Groenenborgerlaan 171, 2020 Antwerp, Belgium; sarah.lebeer@uantwerpen.be; 3 Swammerdam Institute for Life Sciences, Faculty of Science, University of Amsterdam, Science Park 904, 1098 XH Amsterdam, The Netherlands; j.zhang6@uva.nl; 4 Tytgat Institute for Liver and Intestinal Research, Amsterdam Gastroenterology, Endocrinology and Metabolism, Amsterdam UMC, Location Academic Medical Center, 1105 BK Amsterdam, The Netherlands

**Keywords:** ionizing radiation, colorectal cancer, radioprotection, radiotoxicity, organoid, microbiota, organ-on-a-chip

## Abstract

**Simple Summary:**

Because of the limitations of current in vivo and in vitro models, this review considers a human relevant approach: modeling colorectal radiotoxicity through human-derived organoids and microfluidics. This system can offer a closer representation of the microenvironment. Co-culturing bacteria and patient derived tumor organoids under a radiotherapy setup, can enable an understanding of the interplay between radiotherapy, the gut microbiota, patient outcomes and assess radioprotective agents of interest. However, challenges in model development highlight the necessity for refinement, questioning their potential to bridge the gap between preclinical research and clinical applications in CRC treatment.

**Abstract:**

Radiotherapy is a commonly employed treatment for colorectal cancer, yet its radiotoxicity-related impact on healthy tissues raises significant health concerns. This highlights the need to use radioprotective agents to mitigate these side effects. This review presents the current landscape of human translational radiobiology, outlining the limitations of existing models and proposing engineering solutions. We delve into radiotherapy principles, encompassing mechanisms of radiation-induced cell death and its influence on normal and cancerous colorectal cells. Furthermore, we explore the engineering aspects of microphysiological systems to represent radiotherapy-induced gastrointestinal toxicity and how to include the gut microbiota to study its role in treatment failure and success. This review ultimately highlights the main challenges and future pathways in translational research for pelvic radiotherapy-induced toxicity. This is achieved by developing a humanized in vitro model that mimics radiotherapy treatment conditions. An in vitro model should provide in-depth analyses of host-gut microbiota interactions and a deeper understanding of the underlying biological mechanisms of radioprotective food supplements. Additionally, it would be of great value if these models could produce high-throughput data using patient-derived samples to address the lack of human representability to complete clinical trials and improve patients’ quality of life.

## 1. Introduction

Colorectal cancer (CRC) is the third most prevalent cancer worldwide [1,2,3] with nearly 1 million deaths per year, according to the WHO [2]. As a part of treatment optimization, radiotherapy is one of the most used treatments to manage CRC [4]. However, radiotherapy treatment is limited by its toxicity to healthy tissues and potential to induce resistance mechanisms in tumor cells. Therefore, there is growing interest in identifying and applying radioprotective agents that can enhance the effectiveness of radiotherapy while minimizing the toxicity to healthy tissues. The identification of such radioprotective agents is not straightforward since current research does not give a full understanding of their potential. Emerging studies have focused on using microphysiological systems with a radiotherapy setup that models the human cancer microenvironment [5] and that could be used to assess radioprotective candidates. This review explores the use of microphysiological systems in the context of radiotherapy treatment for colorectal cancer and their potential for assessing radioprotective candidates.

## 2. The Use of Radiotherapy and Its Targets

Since their discovery by Röntgen in 1895 and their first clinical use in 1896 [6], X-rays have been widely used in radiotherapy as an effective cancer treatment [7]. Through ionizing molecules, ionizing radiation transfers energy to targeted cells to generate lethal lesions [8]. Treatment relies on the therapeutic ratio that depends on the radio-responsiveness of the tumors [9,10]. This necessitates finding a balance between maximizing the radiation dose to kill cancer cells and minimizing the radiation dose to healthy cells. Various factors, such as linear energy transfer, total dose, fractionation rate, and radiosensitivity, must be considered to modulate the effectiveness of tumor cell killing in the selected treatment strategy [9]. The “5Rs” of radiotherapy, used to describe how the patient’s tumor responds to radiation [11,12], are also essential considerations. They are defined as (1) repair: the ability to repair DNA damage caused by radiation, (2) repopulation: the extent of cell proliferation varying between fractions, (3) redistribution: radiation mostly affects cells in mitosis, while S-phase cells are mostly resistant, (4) reoxygenation: radiation sensitivity increases in oxygen-rich environments, and (5) radiosensitivity: individual radiosensitivity and intrinsic tumor radiosensitivity determine the response to radiation.

**Figure 1:**
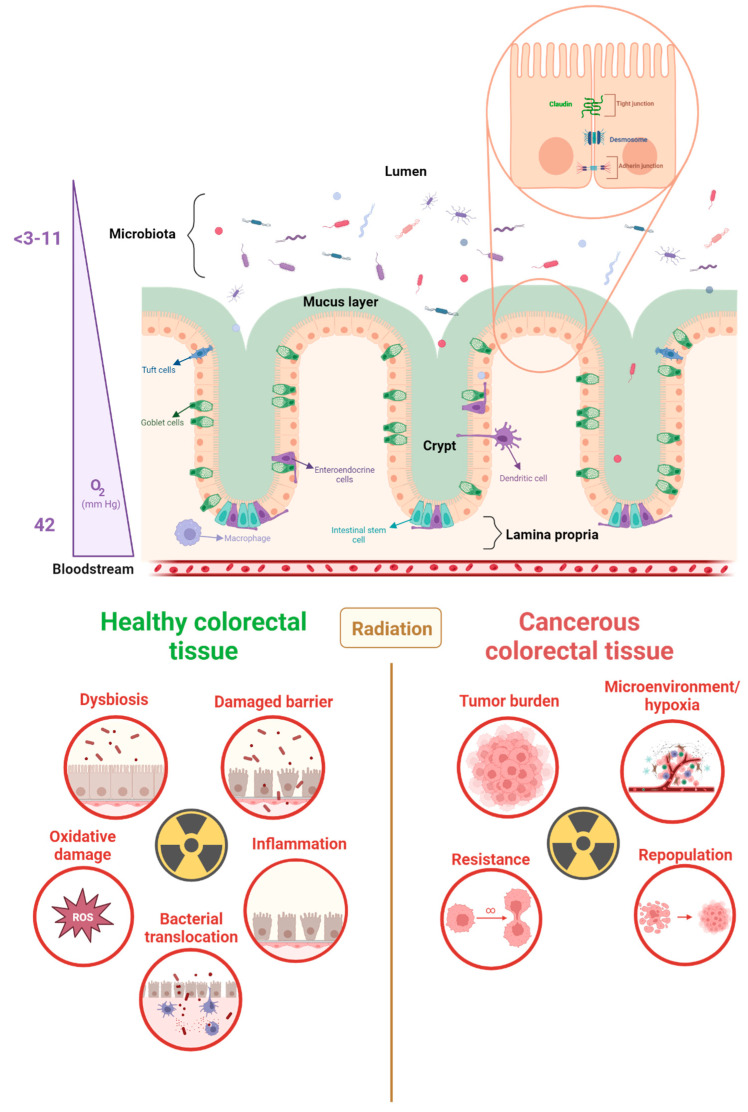
Large intestine morphology, radiotoxicity, and cancer radioresistance. The large intestine exhibits a unique physiology that facilitates its absorption and waste storage functions. The epithelial barrier, which separates the lumen and the lamina propria, is linked by junctions and is covered by a mucus layer. The oxygen gradient within the large intestine, ranging from 3 to 40 pO2 (mmHg), creates an anaerobic environment in the lumen and an aerobic environment in the lamina propria. During radiotherapy, both healthy and cancerous colorectal tissue can be adversely affected by radiation. Radiotoxicity of healthy tissues is mediated by a series of interconnected events, while radioresistance in cancer tissues can result in an increase in tumor growth. This can be attributed to a facilitated microenvironment and tumor-enhancing mutations [20,21,22,23].

Ionizing radiation ruptures chemical bonds when interacting with biological materials. In cells, the main components including proteins, lipids, and nucleic acids can be damaged [13]. DNA is a biological target for radiation because of energy transfer, with single-strand and double-strand breaks affecting tumor cell viability [14]. When double-strand breaks cannot be repaired due to reduced repair capacity, it leads to cell death [15]. Single-strand breaks can be repaired by cells, given the available antisense template. Cancer cells generally produce more double stranded DNA breaks and are less effective in repairing the damage caused by radiotherapy [15]. However, treatment resistance can occur. A consequence of DNA breaks can be an increase in the number of mutations within the DNA of cancer cells. This provokes more proliferation, (for example, alteration in NRAS, KRAS, APC [1]) resulting in tumor burden. Another adverse impact of radiotherapy is the creation of a microenvironment/hypoxia in favor of the tumor, allowing further growth and promoting tumor progression [1]. Concurrently, in the aftermath of irradiation, a phenomenon known as irradiation repopulation takes place, marked by the proliferation of resilient cancer cells. This process is closely intertwined with resistance to treatment, consequently promoting the advancement of cancer cells (Figure 1). This correlates with the importance of the 5Rs in setting up a treatment strategy that can affect cancer cells and avoid resistance. In addition, radiation stimulates the immune system via two main pro-inflammatory pathways: nuclear factor kappa-light-chain-enhancer of activated B cells (NF-κB), and signal transducers and activators of transcription (STAT). NF-κB regulates the expression of anti-apoptotic proteins, pro-inflammatory cytokines, and chemokines such as tumor necrosis factor alpha (TNF-α) and Interleukin-1 (IL1) in macrophages. STAT proteins also play a significant role in tumor development [16]. Among these proteins, STAT3 is associated with radioresistance. As the most important regulator of survivin (part of the apoptosis protein family), its inhibition showed to increase CRC apoptosis in vitro [2]. The STAT1 protein has dual functions, it can induce pro-apoptotic genes, such as *caspase 3*, and promote radioresistant cancer cell phenotypes and tumor metastasis. Additionally, tumor cells and tumor-infiltrating lymphocytes produce cytokines and growth factors (e.g., TNF-α and IL6) in response to radiation and are mostly dose-dependent [17]. These pro-inflammatory responses after radiotherapy, involving the acquired immune system, could create a favorable environment for the tumor through the over-stimulation of inflammatory components, thereby enhancing cancer cell invasiveness [16,17,18,19], which causes unwanted side effects.

## 3. The Radiotoxicity of the Gastrointestinal Tract

Within the context of radiotherapy treatment, it is crucial to consider radiotoxicity, which encompasses the detrimental impact of radiation on healthy tissues. About 90% of pelvic cancer patients in Europe reported gastrointestinal problems during and/or after treatment, with 50% experiencing symptoms such as diarrhea, cramping, and nausea [19,24]. Because of these symptoms, patients need to break from therapy, reducing the effectiveness of the treatment.

### 3.1. The Physiology and Function of the Gastrointestinal Tract

**Figure 2:**
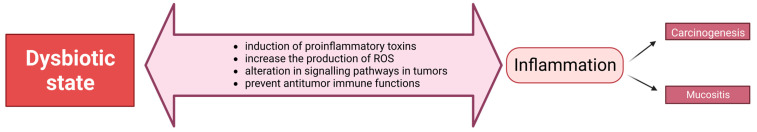
The effect of pathogenic bacteria in dysbiosis. An overgrowth of pathogenic bacteria in the lumen leads to dysbiosis. This has detrimental effects on health through various mechanisms, including the stimulation of an inflammatory state. The resulting mucositis and carcinogenesis can further propagate dysbiosis, leading to an increase in pathogenic bacterial presence in the gut [33]. ROS: reactive oxygen species.

The human digestive system is made of the mouth, pharynx, esophagus, stomach, and the small and large intestines. The gastrointestinal tract is a tubular structure composed of a lumen, mucosa, submucosa, muscularis, and serosa, with different microphysiological and cellular organization in the different parts of the digestive system [21,22]. The small intestine comprises three segments, namely, the duodenum, jejunum, and ileum, which are primarily involved in breaking down food, absorbing nutrients and water, and propelling food through the gastrointestinal tract. In contrast, the large intestine consists of the cecum, colon, rectum, and anal canal. It serves as an organ for absorption, the storage of waste, and the transportation of solid waste. Unlike the small intestine, which has villi and crypts, the epithelium of the large intestine comprises only crypts that allow for interactions with the gut microbiota, thereby creating a compartment for waste storage [21,22,23]. The bottom of the crypts is made of stem cells, that self-renew after 3 to 4 divisions before differentiating and moving up the crypt [25]. Owing to its unique microphysiology, the gastrointestinal tract undergoes renewal every five days [26]. The inner surface of the large intestine is composed of epithelium containing (1) epithelial cells that allow the absorption of nutrients, (2) chemosensory tuft cells that track the luminal gut content and contribute to the immune responses, and (3) goblet cells that secrete mucus to cover the surface of the epithelium (Figure 1). The mucus and intestinal epithelial cells serve as a barrier that prevents bacteria, located in the lumen, from infiltrating the body. Moreover, desmosomes, adherens junctions, and tight junctions allow physical protection and an interface for the immune system (e.g., resident macrophages, dendritic cells, neutrophils, lymphocytes, and monocytes) to induce adequate immunological responses [27] (Figure 1). Therefore, they play a crucial role in the barrier function and exhibit robust adhesion of the epithelial lining [28]. Indeed, the mucosal surface lining regulates its immune responses through mucosa-associated lymphoid tissue (MALT). More specifically in the gastrointestinal tract, gut-associated lymphoid tissues (GALT) induce adaptive immune responses (lymphocytes B and T) [29]. Finally, a specific aspect of the physiology of the gastrointestinal tract is a highly vascularized lamina propria of the intestinal mucosa. It contrasts with an anaerobic lumen inhabited by trillions of metabolically active microbes, creating a very specific tissue microenvironment. Among the microorganisms colonizing the gut, the anaerobic population mirrors the need for hypoxic conditions in the gut environment [30]. The combination of variations in blood flow, epithelial metabolism, and oxygen diffusion into the lumen leads to this particular physiology. Microbiota-produced metabolites, such as short-chain fatty acids (SCFAs), are used by colon epithelial cells and stimulate oxygen consumption via oxidative phosphorylation, sustaining and contributing to the hypoxic environment. By coordinating numerous elements of the intestinal epithelium’s activity (Figure 2), SCFA producers appear to be crucial for preserving physiological hypoxia characterized by a PO_2_ value below 10 mmHg (Figure 1) [30,31,32].

### 3.2. Pelvic Radiation Disease

With the specific physiology we described, in the frame of cancer survival, the rise of research towards treatments and their side effects [34,35,36,37,38,39] has put forward the symptoms of pelvic radiation disease [40,41]. Due to the quick turnover of the gut epithelium, the stem cells present in the small intestine are highly radiosensitive [40,42,43,44,45], resulting in damage to the gut epithelium. There, DNA strand breaks provoke the activation of transcription factors, leading to the release of pro-inflammatory cytokines and chemokines [46]. The production of these mediators stimulates the recruitment of neutrophils, characterizing acute inflammation. This inflammatory process leads to the migration of monocytes, the activation of mast cells produces pro-inflammatory and pro-fibrotic mediators, such as the transforming growth factor beta 1 (TGF-β1), cooperating with IL4 and IL13 cytokines [16,46]. In addition, as ionizing radiation affects the mucus layer and intestinal walls, bacterial translocation occurs and activates inflammatory responses as well. Bacterial toxins that pass through the intestinal wall and enter the bloodstream increase the risk of local infection and sepsis [47].

In parallel, the biological effects on neighboring cells that are not directly irradiated are known as bystander effects or non-targeted responses. They originate from lesions caused by the initial radiation and differ from the alterations that remain after repair. Bystander effects can be classified into two types. One type, called direct bystander effects, involves the release of molecular factors by cells through gap junction intercellular contact [48,49]. In contrast, another type, known as indirect bystander effects, occurs when cells that are further away encounter secreted factors through the bloodstream. The complex mechanisms underlying bystander effects involve various signaling pathways, including oxidative stress, DNA damage, inflammation, apoptosis, and pyroptosis. These pathways activate different molecular factors such as cytokines, growth factors, and reactive oxygen species (ROS). They initiate a series of events leading to biological alterations in neighboring cells. The innate immune system is triggered by damage-associated molecular patterns (DAMPs) and pathogen-associated molecular patterns (PAMPs). These signals primarily recruit resident macrophages and dendritic cells. The cytosolic multiprotein complex, known as the inflammasome, recognizes PAMPs and DAMPs through pathogen recognition receptors (PRRs) and facilitates the proteolytic cleavage of inflammatory proteins in response to endothelial cell proliferation [50]. Furthermore, lipopolysaccharides (LPSs), present on the outer membrane of all Gram-negative bacteria, induce inflammatory responses through Toll-like receptor 4 (TLR4) recognition. This activates the NF-κB pathway resulting in inflammation [51]. However, when LPSs are sensed intracellularly, it stimulates pyroptosis. This pro-inflammatory programmed cell death releases inflammatory components (cytokines, DAMPs, and PAMPs) [52]. Linked through the AIM2 inflammasome, epithelial cell death is mediated, and intestinal radio-sensitivity is regulated through caspase-1 mediation [53]. Under such biological effects, healthy tissues are, together with cancer cells, negatively impacted by the progression and chronicity of radiation injury to the intestinal wall. This can also be facilitated by excessive and chronic ROS and reactive nitrogen species (RNS) generation [46].

Bystander effects have significant implications for radiotherapy as they have the potential to influence treatment efficacy and increase the risk of adverse effects on normal tissues and an elevated risk of secondary malignancies. However, these effects may enhance the elimination of tumor cells by amplifying the extent of tumor damage. Hence, a comprehensive understanding of bystander effects’ mechanisms and the development of strategies to regulate them may lead to better therapeutic outcomes for radiotherapy [13,54,55].

## 4. The Complex Relationship between the Gut Microbiota and the Gastrointestinal Tract

The human gut microbiota is known as the microorganisms that live collectively in the intestinal lumen and mucosal surfaces along the intestinal tract and interact with the different host cells. Studies have shown that the composition of the gut microbiota (notably defined with sequencing methods listing 9,879,896 genes [56]) influences the response to radiotherapy and gastrointestinal toxicity [33]. The different taxa differ in their capacity to modulate innate and systemic immune responses [49,57]. In addition, the members of the gut microbiota possess various physiological capacities required by the host for its functioning, including the biosynthesis of vitamins, steroid hormones, and neurotransmitters [33]; metabolism of xenobiotics [58]; modulation of intestinal epithelial cell turnover [59,60]; and immunomodulatory functions [61,62,63,64,65]. An imbalance of the proper functioning of the gut microbiota can occur if specific members of the gut microbiota are altered (increase in pathogenic members or decrease in protective commensals by ionizing radiation). Such modifications have been linked to several disorders, like colorectal cancer and inflammatory bowel disease [35,66,67,68]. This so-called dysbiosis state highlights the distinction between the impact of commensal microbiota, which plays a role in enteric immunity by shaping the development of the immune system and its responses, [68,69] and pathogenic bacteria. If not controlled by the commensal microbiota, the latter can take over and induce detrimental effects (Figure 2). Examples of this control include the production of inhibitory substances (secondary bile acids) or the consumption of limited common resources (carbohydrates), leading to the starvation of competing pathogens [70]. By stimulating inflammation, certain bacterial species, such as *Fusobacterium nucleatum*, can promote CRC initiation and progression (Figure 2) [33,71,72]. Another study modelled the interaction between a gut microbiota bacterium and the host through intestinal colonization with the microinjection of the non-pathogenic strain *Escherichia coli* ECOR2 into the lumen of human intestinal organoids (HIOs) [73]. The observed stimuli given by the bacterial contact demonstrated an increase in barrier functions (induction of mucus secretion, increased cell junction expression, measure of bacterial translocation), antimicrobial defense (secretion of antimicrobial peptides) and tissue maturation (analysis of principle component to determine tissue maturation status) [73]. Moreover, pathogenic *Clostridium difficile* strain VPI 10463 being microinjected in HIOs, showed to damage the epithelium with impaired barrier function [74].

Those studies reported the impacts that bacteria can have on the host. In the event of radiotherapy, the disruption of the gut microbiota and intestinal tissue can be escalated by each other. Indeed, exposure to radiation results in inflammation leading to gut microbiota dysbiosis that can also cause and influence inflammation in return [75]. This is connected to post-irradiation tissue damage and the pathogenic impact of specific bacteria in the microbiota [42,76]. Dysbiosis of the gut microbiota has been shown to make patients more susceptible to radiation damage and plays a role in aggravating intestinal inflammation (Figure 1) [42,75,77].

### 4.1. The Influence of the Gut Microbiota on Colorectal Cancer

CRC has direct contact with the gut microbiota as it originates from growth on the inner lining of the large intestine. The build-up of numerous independent genetic changes, such as mutations of the *APC*, *TP53*, *KRAS*, *PIK3CA*, *FBXW7*, *SMAD4*, *TCF7L2*, and *NRAS* genes, leads to cancer development. These genes affect cell differentiation, proliferation, and apoptosis in cancer [78]. Aberrant proliferation results in the formation of polyps that can become malignant with the invasion of cancer cells into the submucosa [79,80]. The immune responses of the gut microbiota and host immune responses are linked through the inflammasome, as mentioned before in the radiotoxicity section. Recent research has highlighted the significance of the “inflammasome-microbiota axis” in several disease scenarios, including CRC [81], as the metabolism of the gut microbiota can have both positive and negative effects. In the case of dysbiosis, pathogenic strains can become prevalent over commensal strains. Pathogens such as Escherichia coli can, for example, produce B2-colicin which can induce DNA damage [82,83]. With this role in carcinogenesis, some strains have also been shown to overstimulate immune responses, such as *Bacteroides fragilis* via T helper 17 cells and *Peptostreptococcus anaerobius*, which notably modulates tumor-associated macrophages. The latter strain also interacts with colon cells via TLR2 and TLR4 [71].

### 4.2. Interactions between Bacteria and the Tumor Microenvironment

Solid tumors are sophisticated organs to which numerous other cells are attracted and diverted for their own benefit. The tumor microenvironment (TME) is a cellular environment created by the interactions between malignant and unaltered cells via the lymphatic and circulatory systems to affect the progression of cancer [84]. Its extracellular matrix is composed of various proteins including collagen, fibronectin, elastin, and laminin. This matrix serves as a physical scaffold for cells and can also contain cytokines and growth factors, such as proangiogenic factors including vascular endothelial growth factor (VEGF) and TGF-β. Together, these components not only provide physical support but also promote tumor progression [85].

In the TME, healthy cells play a crucial role in all stages of carcinogenesis by allowing and promoting unchecked cell proliferation [86]. Cancer-associated fibroblasts (CAFs) were notably linked to colorectal cancer recurrence, by producing growth factors, cytokines, and extracellular matrix [87]. Due to the vast heterogeneity of cell types (e.g., T cells, macrophages, CAFs [84]), this microenvironment has a huge impact on how tumors react to treatment [88]. As change in the gut microbiota’s balance is systematically observed in patients with colorectal cancers, it has the potential to alter the TME and create a favorable environment for tumor growth [89]. Bacteria that live inside tumors have been found to be tumor type-specific, indicating a connection with tumor progression by increasing mutagenesis, regulating oncogenic pathways, and modulating the host immune system [90]. The latter is notably witnessed in CRC tissues, with *Fusobacterium nucleatum* that provides a pro-inflammatory environment by activating the NF-κB pathway [91]. It also modifies the TME to evade anticancer immune responses by binding to fatty-acid-binding protein 2 (Fap2), an adhesin that binds to natural killer cells and other tumor-infiltrating lymphocytes via T-cell immunoglobulin (TIGIT) receptors [92].

## 5. Using Food Supplements to Mitigate Radiotoxicity

As radiation toxicity is a major concern for cancer patients undergoing radiotherapy, it is leading researchers to focus on the prevention and mitigation of its effects, notably using radioprotectors. There are currently only two radioprotective agents approved by the Food and Drug Administration: palifermin (Kepivance^®^) and amifostine (Ethyol^®^). The former is an artificial keratinocyte growth factor used to diminish severe oral mucositis. One of the potential concerns of its use regards its mitogen impact on epithelial cells which could promote tumorigenesis [3]. On the other hand, amifostine is an inactive phosphorylated aminothiol prodrug. It is dephosphorylated by alkaline phosphatase to its active and free radical-scavenging sulfhydryl metabolite: WR-1065 [93]. It can selectively protect healthy tissues, such as the intestine, but may cause side effects, such as nausea and diarrhea, as well as allergic reactions [94]. In addition, its potential to also protect malignant tissues from radiotherapy still needs clarification [95]. To avoid these side effects and reduce injury to healthy intestinal tissues, researchers have investigated the use of bacterial food supplements instead, as safer radioprotectors including probiotics and prebiotics [96,97] They have been the primary focus of previous in vitro and in vivo research on radioprotection mechanisms, with antimicrobial, barrier-enhancing, and immunomodulatory capacities being the most extensively studied functions [57,71,96]. Intestinal cells treated with specific probiotics or prebiotics before and/or after radiation, have already suggested the potential of their action [97,98,99], but remain to be confirmed by dedicated clinical trials. In 2021, Tripathy et al. reviewed the impact of several probiotic strains on colorectal cancerous cell lines. For instance, *Lacticaseibacillus rhamnosus* GG was tested on the LT-97, HT-29, HCT-116, and Caco-2 cancer cell lines. This model probiotic strain is shown to stimulate apoptotic pathways in malignant cells, halt their proliferation, enhance the release of interleukin-8 (a chemokine that attracts immune cells including neutrophils), and modulate SCFA production [28]. As the product of microbial fermentation, the latter offers protective benefits to healthy cells by enhancing barrier function through tightening junctions and alleviating metabolic stress [28]. Colonocytes absorb these molecules and upregulate the expression of apoptosis-activating proteins in CRC cells [28]. They are essential as they restrict cancer tissue growth by modulating cell differentiation and proliferation through the regulation of cyclin expression at cell cycle checkpoints [100]. The retinoblastoma protein, a tumor suppressor, notably targets D-type cyclin as a substrate. Therefore, this protein enables cells in the G0 phase to progress to the G1 phase and regulates its progression. Moreover, A-type cyclin is also important as it participates in DNA synthesis but also helps prevent its overabundance by facilitating entry into the S phase, completion of the S phase, and entry into the M phase [100]. This highlights that proper microbial fermentation through commensal microorganisms, producing SCFAs, is essential.

The use of specific probiotics and prebiotics to complement CRC treatment has also been studied in clinical trials, showing evidence of their efficacy on the quality of life of patients experiencing fewer symptoms and lowered inflammatory marker concentrations, such as TNF-α levels [101,102]. Clinical trials recapitulated by Shuwen et al. showed that SCFAs, from microbial fermentation, have been used as therapeutic treatment against CRC cells [5]. One positive impact is the exertion of a regulatory influence on the immune response by promoting the production of anti-inflammatory cytokines [103] In 2016, Mansouri-Tehrani et al. reported the impact of probiotics alone, coupled with honey, and placebo controlled in 67 patients with pelvic cancer receiving radiotherapy, who were exposed to a total dose between 40 and 50 Gy. Species including *L. casei*, *L. rhamnosus*, and *Bifidobacterium breve* were combined and administered orally before and during radiotherapy. The Food and Drug Administration and Isfahan University of Medical Sciences evaluated and authorized the use of probiotics and honey in this study [104]. Another clinical trial by Delia et al. regrouped 490 sigmoid, rectal, and cervical cancer patients following postoperative radiotherapy treatment with radiation doses within the range of 60–70 Gy with a combined solution, administrated orally, of the following probiotics: *L. casei*, *L. acidophilus*, *L. rhamnosus*, *L. bulgaricus*, *B. breve*, *B. longum*, and *S. thermophiles* [104]. Patients in these clinical trials showed a reduced incidence of radiation-induced diarrhea and improved quality of life.

In parallel, studies testing the use of prebiotics showed their impact on colon cancer cells. Arun et al. reported that the fermentation (by probiotics) of the plantain inflorescence dietary fiber produces SCFAs and induces apoptosis of HT29 cells [105]. Moreover, Nowacka-Jechalke et al. studied the role of polysaccharides (prebiotic) from the mushroom *Cantharellus cibarius* in preventing and treating colon cancer [106]. Those metabolites act on inhibiting the proliferation of colon cancer cells and on stimulating *Lactobacillus* strains growth. While the existing literature provides the results of the efficacy of probiotics and prebiotics as adjuncts to radiotherapy, there is still missing data on the beneficial effects because of the low number of clinical trials investigating their use in this context. Furthermore, a significant proportion of the studies to date have focused on using food supplements in the case of chemotherapy rather than radiotherapy treatment. Finally, the heterogeneity of studies with respect to radiation doses, and radiation administration frequency limits the general application of the findings.

## 6. Modeling Colorectal Radiotoxicity with Human Gut In Vitro Models

### 6.1. Radiobiology Models: Pros and Cons

Before reaching human clinical trials, animal testing was abundantly used in radiobiology. Animal research models, regulated by law, government policies, and ethical guidelines, provide a representation of the entire organism through its regulatory processes and responses [107]. Through genetic manipulation and disease induction, in vivo animal models allow the study of radiosensitivity as well as radioresistance [108]. This enables the modeling of conditions of radiotherapy treatment with disease reproduction and the study of the role of specific genes, such as ATM or p53, in radiobiology [109,110], or testing of treatment enhancing strategies such as food supplements [98,111]. In 1959, Russell and Burch introduced the 3Rs, defining the concepts of replacement, reduction, and refinement [112,113]. The goals are to find alternatives, reduce the number of animals used, and reduce the harshness of animal testing. Working with animals to study the interaction of gut microbiota and the gastrointestinal tract also presents a major issue. With its low degree of translation in terms of gut microbiota composition, it provides poor human relevance [114]. Alternatively, in vitro models using human cells can be used to get closer to the human reference. Cell culture plates deliver high-throughput screening, allow genetic manipulation, have the potential to provide a personalized approach, and are easy to handle [113,115]. For radiobiology, in vitro models have been used to complement in vivo preclinical studies with human relevance to test novel irradiation treatments, such as FLASH irradiation [116], optimize the conditions of exposure [117], and evaluate the potential of radiosensitizers [118] or prebiotic supplements [119]. However, cell culture models lack cell–cell interactions, do not give control of in vivo cell morphology, do not give a gut microbiota representation nor microenvironment management, creating a gap with in vivo representation [115,120].

### 6.2. Providing Human Relevance and Representation of Microorganisms-Host Interaction with Microphysiological Systems

**Figure 3:**
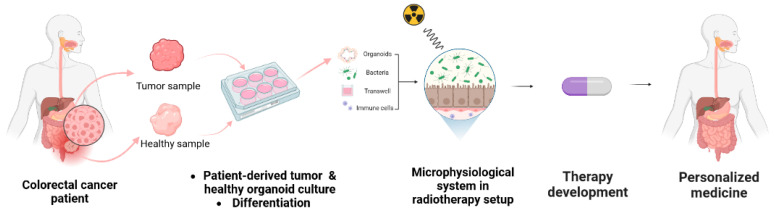
Set up of personalized medicine. Incorporating patient-derived samples into microphysiological systems under conditions mimicking radiotherapy enables the representation of treatment conditions and the development of novel therapies [86].

Owing to these limitations, interest has grown in 3D models coupled with the use of microphysiological systems, which represent the complexity of living tissues more accurately [120,121]. In human-specific studies, the use of organoids, 3D organ-like models, allows for the adoption of a translational human approach [122]. To represent large intestine tissues, colorectal organoids contain differentiated enterocytes, goblet cells, and tuft cells, and can recapitulate developing crypts and mucus layers [123]. They can be co-cultured with immune cells to give a better microenvironment representation [124]. To mimic the responses of patients to treatment-induced toxicity [125,126,127,128,129], patient-derived tumor organoids (PDTOs) [130] can be used to provide an improved model to study the effects of irradiation on colorectal tissue and gut microbiota (Figure 3). With radiotherapy and PDTOs being an increasing focus of studies since 2018 [131], multiple models derived from rectal cancer patients undergoing radiotherapy have been implemented to understand resistance mechanisms to treatment, predict treatment outcomes, and evaluate new adjuvant treatment strategies, including food supplements [128,129,132,133,134]. However, organoids have a wide range of sizes and shapes, making it challenging to keep cells in stable positions in these structures for prolonged examination and to sample for analysis. Furthermore, limitations are posed with PDTOs being cultured with different protocols and in different conditions, creating variability in the existing studies [135]. Tissue–tissue interactions and multiscale architectures are absent in many systems, and cells are not typically exposed to physiological mechanical cues, such as fluid shear stress, tension, and compression. The latter affects how organoids grow and function in both health and disease, suggesting that supplying these elements via microfluidic technology may increase representativeness and human relevance [121].

### 6.3. Implementing Microfluidic Technology

Radiobiology requires complex models to allow representation at both tissue and organ levels. Various cell types, their interactions, and the release of cytokines, growth factors, and chemokines are necessary to model radiation-induced toxicity, tumor radioresistance, and the interaction with the microenvironment [136]. Microfluidics allows for culturing in continuously perfused chambers and provides physiological models. They represent the function of targeted tissues, recreate the interface between different tissues, and provide cell–cell interactions. This technology can be used to maintain tissue structure and function [121]. Its features allow control of the microenvironment with the laminar flow of the culture medium and biocompatibility between the cultures and the material used [137]. Channels, valves, reservoirs, membranes, and other microscale fluid-handling compartments constitute a microfluidic platform. This enables integrated, automated, parallel, and miniaturized biochemical analyses in a consistent manner [115]. It allows representation of in vitro radiotherapy [5], the co-culture of epithelial cells with bacteria [138,139], the modeling of cancer [140,141], drug discovery with toxicity tests [142], and the study of the barrier function [143].

Such systems can be coupled with PDTOs to establish microphysiological systems. The ideal gut microphysiological model, described in Table 1 (first column), mimics the structure, function, and physiology of the human intestine and its interaction with microorganisms from the gut microbiota. The use of organoids to form monolayers provides in vitro intestinal epithelium representation and access to both the luminal and basal sides. Recreating the oxygen gradient is necessary to allow co-culture of oxygen-sensitive microorganisms and primary colorectal epithelial cells. To simulate the lumen environment described previously, e.g., *Bifidobacterium* and *Lactobacillacea* species (frequently employed as food supplements or probiotics as described above) require anaerobic conditions on the apical side. While the basolateral side requires aerobic conditions to mimic the oxygenated blood-rich lamina propria environment that contains resident immune cells. The hardware design allows fluid pumping to create apical and basolateral flow to model shear stress, respectively, remove spent medium, promote oxygenation, and supply oxygen needed for the epithelial cells [139]. To support cell attachment, a specific scaffold material that remains intact, compatible with radiation, and that does not absorb compounds is essential. However, considering the sensitivity of the host cells to living bacteria and their metabolites remains crucial. In 2011, Sato et al. published the first description of the long-term culture of epithelial organoids made from human colorectal and neoplastic tissues [144]. Another specificity that in vitro microphysiological systems can provide is the TME, as described previously. Growing PDTOs from a patient’s colorectal cancer sample coupled with a microfluidic system (Figure 3) allows for the representation of colorectal cancer cell subtypes and their microenvironments [86,123]. This technology is particularly useful for precision medicine by enabling the use of patient samples (Figure 3) [122]. These cancer organoid (i.e., tumoroid) models from primary patient tumor cells can allow the study of treatments and their efficiency [145], by reproducing the contact of the epithelium with microorganisms. This raises the question of whether combining a microphysiological system with a radiotherapy setup can provide representative treatment conditions. Indeed, radiotherapy treatment presents challenges in its establishment with the timing, irradiation dose, dose rate, and fractionation. Such a representation would allow the study of the effect of radiation on colorectal cancer tissue and new personalized treatment strategies such as the use of food supplements [136].

### 6.4. Radiotherapy Setup for the Evaluation of Novel Treatments

The choice of cell type and the in vitro microenvironment is crucial for achieving an ideal model that closely mimics in vivo tissue. Using PDTOs instead of immortalized cancer cell lines, can preserve tissue characteristics that are otherwise lost. In addition, accurately controlling oxygen levels allows for the aforementioned gradient of the colorectal cancer environment to be replicated. To show the human relevance of tumoroids, Vlachogiannis et al. compared PDTOs from 71 colorectal cancer patients to parental tissue biopsy, showing significant similarities in the pattern of tumor expression such as the responsiveness to treatment [145]. Current models (Table 1) including both human immortalized cell lines and microbial cells enable maintaining this kind of co-culture for up to seven days using biocompatible hardware materials. The latter also need to be suitable for the type of study of interest. In radiobiology studies, the hardware must be resistant and compatible with ionizing irradiation. Previously used in human gut models, (Table 1) polydimethylsiloxane (PDMS) has many useful physical properties. However, it showed that it absorbs hydrophobic compounds such as pharmaceutical compounds (ciprofloxacin and paclitaxel) [158]. This can have a detrimental impact on the biological functioning and measurements by modifying the microenvironment composition by altering the concentrations of essential components of the cell culture (amino acids, growth factors, vitamins, etc.). Other materials such as monolithic polysulfone, that have been shown to be inert, seem to be more suitable [146].

To study the impact of radiotherapy on the gut and its microbiota, a model that provides oxygen control with both apical anaerobic conditions for bacteria, and basolateral aerobic conditions for epithelial and immune cells has been developed [147]. That complete system could enable the generation of high-content data on the testing of various food supplements type and concentration to prevent radiation induced toxicity (Figure 3). Further studies on microphysiological systems would provide more data to support their implementation by pharmaceuticals industries.

## 7. Conclusions

As radiotherapy is a pillar of CRC treatment, counteracting its radiotoxicity remains a priority. Research is increasingly exploring ways to do so through in vivo, in vitro, and clinical studies. A central point of focus within this field revolves around investigating the radioprotective mechanisms of food supplements. Evidence of their beneficial impact on health by balancing the gut microbiota, enhancing barrier function, and immunomodulation has been observed. Human derived organoids have emerged as innovative 3D tools for studying human biology and disease modeling to address the limitations of existing in vitro cell lines and animal radiobiology models in terms of human relevance. Coupling them with microfluidics to design microphysiological systems can provide a representation of cellular components and microenvironments. To assess radioprotective candidates, such as food supplements, there is a need to provide human-relevant bacteria–host co-culture representation under radiotherapy treatment conditions. This model of gut radiotoxicity offers a deeper mechanistic insight. Given the significant role of the microorganisms of the gut microbiota in modulating systemic immune responses that affect patient outcomes, combining tumor organoids with such a model exposed to radiotherapy presents a promising opportunity. However, the development of these models also comes with limitations. The establishment of organoid protocols lacks standardization, which may lead to variability in study outcomes. Furthermore, microphysiological models of the human gut have yet to implement the immune system and the gut microbiota. Also, by only featuring differentiated cells, the replacement of dead cells by stem cells and the bystander effect are not modeled. Still, using the described model, research would provide more human-relevant data, which could guide further translation in human patients by performing clinical trials.

## Figures and Tables

**Table 1:** Overview of gut microphysiological models compared to the ideal human relevant model.

Models	Ideal Gut Microphysiological Model ^†^	GuMI [146,147]	EACC [148]	HuMix Model [149] HMI Module [150]	Gut Chip [151,152]	AOI Chip [153]	Nicole C. Roy [154]	HoxBan [155]
Cell types	Epithelial cells, goblet cells, innate immune cells, adaptive immune cells, bacterial cells	Primary colon epithelial cells	Human primary jejunal enteroids (4 donors)	Caco-2 cells; CCD-18Co	Caco-2 cells/primary cells	Caco-2BBE cells	Caco-2 cells	Caco-2 cells
Cell architecture	Monolayer with villus shape	Monolayer	Monolayer	Monolayer	Microfluidic chip	Microfluidic chip	Monolayer	Monolayer
Barrier function	Mucus, TEER measurements close to the in vivo: 300–400 Ω cm^2^ in the large intestine [156]	Yes, TEER measurements significantly higher than 300 Ω cm^2^	Yes, only expressed in fold changes in TEER. Increase after bacteria exposure	TEER measurements, 1000 Ω cm^2^	TEER measurements > 2500 Ω cm^2^	TEER measurements: 5–10 kΩ cm^2^	60% decrease (even in control) in first 1 h, then recover.	No values, cells attached to glass slide
Contact of bacteria and host cells	Direct contact or separate by mucus layer	Yes	Yes	No, separated by membrane	Yes	Yes	Yes	Yes
Anaerobic conditions (oxygen level)	Yes, ideally gradient anaerobic conditions	Anaerobic, low oxygen content	Yes	0.1% O_2_; oxygen optode sensors measurement	Yes	Yes	Yes	Yes
Co-culture maintaining time	Yes, ideally one week or more *	5 days	8–24 h	Up to 2 days	Up to 5 days	Up to 7 days	Half a day	Up to 1 day and a half
Static or flow	Flow, in both apical and basal sides	Flow, 600 µL/h	Static	Flow, 1500 µL/h [0.416 µL/s]	Flow, 30 µL/h [0.05 µL/min]	Flow, 50 μL/h	Static	Static
Immune cells	Yes, both innate and adaptive immune cells	dendritic cells, macrophages, CD4+ T cells	No	CD4+ T cells in third layer	PBMCs	No	No	No
Bacterial species	Oxygen sensitive strain	*Faecalibacterium prausnitzii*, *Eubacterium rectale*, *Bacteroides thetaiotaomicron*	*B. thetaiotaomicron* and *Blautia* sp.	*Lacticaseibacillus rhamnosus GG* *Bacteroides caccae*	*E coli*, probiotic mix (*Lactobacillus acidophilus, Lactobacillus plantarum*, *Lactobacillus paracasei*, *Bifidobacterium breve*, *B. longum*, *B. infantis*)	*Bifidobacterium adolescentis* and *Eubacterium hallii*	*F. prausnitzii* DSM17677	*F. prausnitzii*
Hardware material	Biocompatible, intact while running	Monolithic polysulfone (low absorption of hydrophobic compounds)	Not specified	polycarbonate (PC) (low absorption of hydrophobic compounds)	polydimethylsiloxane (PDMS) (high absorption of hydrophobic compounds)	PDMS (high absorption of hydrophobic compounds)	Not specified	Glass for cell attachment and agar for bacteria (low absorption of hydrophobic compounds)
Stress applied (um/s)	Fluid shear stress: ~0–2.5 µPa [157]	Shear stress: 0–11 µPa	Not applied	Not specified	Not specified	Shear stress: 3–10 µPa	Not applied	Not applied

^†^ Based on the gastrointestinal tract physiology. * One week of co-culturing is the target because differentiated cells are used in the model and cannot model the physiological replacement of dead cells by stem cells. TEER = trans-epithelial electrical resistance; PBMC = peripheral blood mononuclear cells.
